# Establishment and application of loop-mediated isothermal amplification coupled with nanoparticle-based lateral flow biosensor (LAMP-LFB) for visual and rapid diagnosis of *Candida albicans* in clinical samples

**DOI:** 10.3389/fbioe.2022.1025083

**Published:** 2022-11-07

**Authors:** Yu Wang, Xue Zhao, Yuhong Zhou, Jingrun Lu, Honglan Yu, Shijun Li

**Affiliations:** ^1^ Department of Clinical Laboratory, The First People’s Hospital of Guiyang, Guiyang, China; ^2^ School of Public Health, The Key Laboratory of Environmental Pollution Monitoring and Disease Control, Ministry of Education, Guizhou Medical University, Guiyang, China; ^3^ Laboratory of Bacterial Infectious Disease of Experimental Center, Guizhou Provincial Centre for Disease Control and Prevention, Guiyang, China

**Keywords:** Candida albicans, loop-mediated isothermal amplification, lateral flow biosensor, LAMP-LFB, limit of detection

## Abstract

*Candida albicans* is an opportunistic pathogenic yeast that predominantly causes invasive candidiasis. Conventional methods for detecting *Candida* species are costly, take 3–5 days, and require skilled technicians. Rapid pathogen identification is important in managing invasive candidiasis infection. Here, a novel molecular diagnostic assay termed loop-mediated isothermal amplification combined with nanoparticles-based lateral flow biosensor (LAMP-LFB) was developed for *C. albicans* rapid detection*.* A set of six primers was designed based on the *C. albicans* species-specific internal transcribed spacer 2 (ITS2) gene. The *C. albicans*-LAMP results were visually reported by LFB within 2 min. Various fungal strains representing *Candida* species, as well as several Gram-negative and Gram-positive bacterial species, were used to determine the analytical sensitivity and specificity of the assay. The optimal LAMP conditions were 64 °C for 40 min, with a sensitivity of 1 fg of genomic DNA template from *C. albicans* pure cultures. No cross-reactions were obtained with non-*albicans* strains. Thus, the analytical specificity of the LAMP-LFB assay was 100%. The entire procedure could be completed within 85 min, including specimen processing (40 min), isothermal reaction (40 min), and result reporting (within 2 min). In 330 clinical samples (including 30 whole blood, 100 middle segment urine, and 200 sputum samples), all *C. albicans-*positive (62/330) samples were identified by LAMP-LFB assay, and the diagnostic accuracy was 100% when compared to the traditional clinical cultural-based methods. Thus, this assay can be used as a diagnostic tool for the rapid, accurate, sensitive, low-cost and specific detection of *C. albicans* strains, especially in resource-limited settings.

## Introduction

The invasive fungal disease is a neglected disease that threatens public health, is difficult to diagnose with long treatment time, costly, and poor treatment efficacy (Fisher et al., 2020; Rodrigues and Nosanchuket al., 2020). Epidemiological statistics depict that invasive fungi cause more than two million infections yearly, and the number of deaths is equivalent to tuberculosis or malaria (Schmiedel and Zimmerli, 2016). Invasive candidiasis is the most common invasive fungal disease with high morbidity and mortality. Even when the patients are treated with antifungal therapy, the mortality rate is as high as 45%, and approximately 50% of cases are attributed to *C. albicans* (Zilberberg et al., 2008; Dadar et al., 2018; Quindós et al., 2018). *C. albicans* is a symbiotic yeast on the human mucosal surface. Simultaneously, *C. albicans* is an opportunistic pathogen responsible for invasive candidiasis such as pyelonephritis, endocarditis, or candidemia (Sherry et al., 2014). According to several recent studies, biofilm formation strengthens *C. albicans* drug resistance and the immune system (Ben-Ami 2018). Additionally, the emergence of drug-resistant strains such as *Candida auris* is partially attributable to empirical treatment without an early and clear diagnosis (Adams et al., 2018; Eyre et al., 2018). Thus, patients lose the optimum time for treatment leading to increased mortality, and drug-resistant strains become prevalent (Fortún et al., 2012). Therefore, rapid and accurate *C. albicans* detection is critical in the prevention, control, and timely treatment of invasive candidiasis, and it can also prevent the emergence of drug-resistant strains.

Unfortunately, current methods for detecting *C. albicans* infections are inadequate to meet clinical requirements. The traditional culture method is the gold standard for diagnosing fungal infections, but it is time-consuming and insensitive (Pincus et al., 2007). Moreover, *C. albicans* detection is easily confused with *Candida dubliniensis* in some colorimetric methods; *C. albicans* is not always detected accurately and rapidly. Therefore, it is important to establish a rapid and effective method for *C. albicans* detection in grass-roots hospitals. Rapid detection receives considerable attention due to the high morbidity and mortality of invasive candidiasis and the increasing prevalence of drug-resistant candidiasis. In recent years, many methods that are more sensitive and faster than traditional culture have emerged for the identification of *C. albicans* have been developed, such as immunofluorescence (Gunasekera et al., 2015) and PCR-based assay (i.e., multiplex PCR and real-time PCR) (Kasai et al., 2006) and mass spectrometry (Zehm et al., 2012). These methods are unsuitable for popularization, especially in resource-poor areas, because they are complex and require expensive equipment and professionals.

In recent years, various isothermal amplification methods, including loop-mediated isothermal amplification (LAMP), multiple cross displacement amplification (MCDA), and recombinase polymerase amplification (RPA), have been designed for nucleic acid analysis to overcome the disadvantages of traditional culture and PCR-based methods and have been reported to detection of *C. albicans* (Zhao et al., 2019; Fallahi et al., 2020; Wang F. et al., 2021). Among them, loop-mediated isothermal amplification (LAMP) is a nucleic acid amplification technique under isothermal conditions that is simpler, economical, sensitive, and rapidly applied in many fields, especially in molecular medical diagnostics (Notomi et al., 2000; Dolka et al., 2019). For example, the LAMP method has been applied successfully to detect *Staphylococcus aureus*, *C. auris*, and the COVID-19 virus (Wang et al., 2017; Yamamoto et al., 2018; Zhu et al., 2020). Conventionally, LAMP amplification products can be tested using various methods, including agarose gel electrophoresis and turbidimetry changes (Hill et al., 2008; Zhao et al., 2020). However, these methods been restricted due to the need for expensive special equipment (real-time turbidimeter or fluorescence apparatus), expensive regents (calcein), and additional analysis procedure (agarose gel electrophoresis) to indicate assay’s results. Therefore, an economical, rapid, simple, accurate, and suitable method for *C. albicans* detection is needed urgently.

In the past few years, nanomaterial-based biosensors have been widely used for the bioassay detection of a broad variety of targets in specimens from cancer patients (Patel et al., 2022a; Azzouz et al., 2022), cardiovascular disease (Azzouz et al., 2021), metabolites in sweat (Wang et al., 2022), proteins (Patel et al., 2022b), bacteria (Li et al., 2019) and fungi (Wang Y. et al., 2021). In the diagnosis of fungi and bacterial infections, biosensors are extremely accurate and quick (Li et al., 2019; Wang Y. et al., 2021). In the current study, we aim to combine classic Loop-mediated isothermal amplification (LAMP) with the gold nanoparticle-based lateral flow biosensors (LFB) to detect *C. albicans*, which is a new method to detect specific gene fragments of microbial with convenient and visual (Li et al., 2019). Moreover, we evaluated the ability to detect clinical samples with *C. albicans*-LAMP-LFB, to establish a fast, economical, accurate, and convenient tool for *C. albicans* detection in clinical applications.

## Materials and methods

### Reagents and instruments

Fungal and Bacterial genomic DNA extraction kits (Baitaike DNA mini kits, China) were obtained from Baitaike. Co., Ltd (Beijing, China). DNA Isothermal amplification kits, colorimetric indicator (Visual detection reagent, VDR), and biotin-14-dCTP were supplied by Tian-Jin HuiDeXin Biotech. Co., Ltd (Tianjin, China). The LFB materials, including the backing card, sample pad, conjugate pad, absorbent pad, and nitrocellulose membrane (NC), were obtained from Jie-Yi Biotech. Co., Ltd (Shanghai, China). Rabbit anti-fluorescein antibody (Anti-FITC) and biotinylated bovine serum albumin (biotin-BSA) were acquired from Abcam. Co., Ltd (Shanghai, China). Dye (Crimson red) streptavidin-coated polymer nanoparticles (129 nm, 10 mg ml^−1^; 100 mm borate, pH 8.5, with 0.1% BSA, 0.05% Tween 20 and 10 mm Ethylene Diamine Tetraacetic Acid, EDTA) were purchased from Bangs Laboratories, Inc (Indiana, United States).

### LAMP assay primers design and synthesis

**FIGURE 1:**
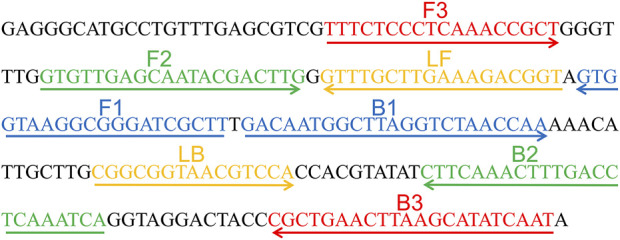
Sequence and location of ITS2 gene used to design loop-mediated isothermal amplification primers. The nucleotide sequences of the sense strand of ITS2 are listed. Right arrows and left arrows indicate sense and complementary sequences that are used.

A set of six primes including two inner primers (FIP and BIP), two outer primers (F3 and B3), and two loop primers (LF and LB), were designed based on the reaction mechanism of LAMP to target the sequence of internal transcribed spacer 2 (ITS2) gene (Genbank accession no. AF455531.1) of *C. albicans* for the LAMP assay. All primers were designed using Primer Explorer V5 (http://primer-explorer.jp/e/; Eiken Chemical Co., Ltd, Tokyo, Japan) and validated using the basic local alignment search tool (BLAST). Figure 1 displays primer positions, while Table 1 depicts the primer sequences and modifications. In this study, all the primers were synthesized by Kunming-Tsingke Biotechnology Co., Ltd (Kunming, China) with HPLC purification grade.

**TABLE1:** Primers used in this study.

Primers name^a^	Sequences and modifications (5′-3′)^b^	Length^c^	Gene
F3	TTTCTCCCTCAAACCGCT	18 nt	ITS2
B3	ATT​GAT​ATG​CTT​AAG​TTC​AGC​G	22 nt	
FIP	AAG​CGA​TCC​CGC​CTT​ACC​AC-GTG​TTG​AGC​AAT​ACG​ACT​TG	41 mer	
FIP*	biotin-AAGCGATCCCGCCTTACCACGTGTTGAGCAATACGACTTG	41 mer	
BIP	GAC​AAT​GGC​TTA​GGT​CTA​ACC​AA-TGA​TTT​GAG​GTC​AAA​GTT​TGA​AG	47 mer	
LF	ACCGTCTTTCAAGCAAAC	18 nt	
LF*	FAM-ACCGTCTTTCAAGCAAAC	18 nt	
LB	CGGCGGTAACGTCCA	15 nt	

^a^ FIP*, 5′-labeled with biotin when used in the C. albicans-LAMP-LFB, assay; LF*, 5′-labeled with FAM, when used in the C. albicans-LAMP-LFB, assay.

^b^ FAM, 6-carboxy-fluorescein.

^c^ Mer: monomeric; nt: nucleotide.

### Fungal and bacterial strains genomic DNA preparation

The 43 fungal and four bacterial strains were employed in the current study (Table 2), including the *C. albicans* reference strain (ATCC10231), 15 isolated *C. albicans*, and 27 non-*C. albicans* fungal strains from clinical samples. All genomic DNA templates were extracted using DNA extraction kits following the manufacturer’s instructions, and the concentration and purity were measured using a Nanodrop 2000 (Thermo Fisher Scientific) at A260/280. The extracted DNA templates were stored at −20°C until further analysis. The genomic DNA of the *C. albicans* reference strain (ATCC10231) was serially diluted to concentrations ranging from 10 ng/μL to 100 ag/μL (10 ng/μL, 10 pg/μL, 1 pg/μL, 100 fg/μL, 10 fg/μL, 1 fg/μL, and 100 ag/μL), to optimize the reaction temperature, reaction time, specificity and sensitivity.

**TABLE 2:** Fungi and bacteria strains used in the study.

Strains	Strain no. (source of strain)^a^	No.of strains	*C. albicans -LAMP-LFB* ^b^
*Candida albicans*	ATCC 10231	1	P
*Candida albicans*	Isolated strains (GFPH)	15	P
*Candida dubliniensis*	Isolated strains (GFPH)	1	N
*Candida tropicalis*	ATCC 13803	1	N
*Candida tropicalis*	Isolated strains (GFPH)	5	N
*Candida krusei*	Isolated strains (GFPH)	5	N
*Candida glabrata*	Isolated strains (GFPH)	5	N
*Candida parapsilosis*	Isolated strains (GFPH)	5	N
*Cryptococcus neoformans*	Isolated strains (GFPH)	1	N
*Penicillium mameffei*	Isolated strains (GFPH)	1	N
*Candida stellatoidea*	Isolated strains (GFPH)	1	N
*Candida guilliermondi*	Isolated strains (GFPH)	1	N
*Aspergillus flavus*	Isolated strains (GFPH)	1	N
*Staphylococcus aureus*	Isolated strains (GFPH)	1	N
*Klebsiella pneumoniae*	Isolated strains (GFPH)	1	N
*Pseudomonas aeruginosa*	Isolated strains (GFPH)	1	N
*Acinetobacter baumannii*	Isolated strains (GFPH)	1	N

^a^ GFPH, The First People’s Hospital of Guiyang; ATCC, american type culture collection.

^b^ P, positive; N,negative. Only genomic DNA, templates from C. albicans could be detected by C. albicans-LAMP-LFB, assay, indicating the extremely high specificity of the method.

### Gold nanoparticle-based lateral flow biosensor preparation

LFB was constructed and used for reporting LAMP results according to the description of a previous publication in our study (Li et al., 2019). Briefly, LFB included an absorbent pad, an immersion pad, an NC membrane, a conjugate pad, and a backing pad. Dye streptavidin-coated polymer nanoparticles (SA-PNPs) were impregnated on the conjugate pad. Then, anti-FITC and biotin-BSA were immobilized at the test line (TL) and control line (CL), respectively. According to our design, the LFB was manufactured timely by Tian-Jin HuiDeXin Biotech. Co., Ltd (Tianjin, China). The LFB was stored in a dry place away from light at a storage temperature of 2°C–25°C, valid for 18 months.

### The standard *C. albicans*-LAMP assay

*C. albicans*-LAMP reactions were conducted per the manufacturer’s instructions for the DNA Isothermal amplification kits. Each reaction mixture contained 12.5 μl of supplied buffer (2×), 0.4 μm of each outer primer, F3 and B3, 0.8 μm of each loop primer, LF* and LB, 1.6 μm of each inner primer, FIP* and BIP, 0.4 mm biotin-14-dCTP, 1 μl (8 U) 2.0 Bst DNA polymerase, and one μ1 DNA template and 1 μl VDR, and distilled water (DW) to a total volume of 25.0 µl. The reaction mixture was incubated for 40 min at 64°C. Moreover, genomic DNA from non-*C. albicans* strains, including *Candida tropicalis* and *Klebsiella pneumoniae,* were used as a negative control (NC), and blank control mixtures contained 1 μl of distilled water (DW). The *C. albicans*-LAMP products were determined and verified using two distinct techniques, including VDR and LFB methods. The color of the amplified products effectively changed from colorless to light green in the VDR assay. However, the negative and blank controls remained colorless. The strategy for visualizing LAMP products with LFB was as previously described.

### Reaction temperature optimization for *C. albicans-*LAMP assay

In this study, the optimal temperature of the *C. albicans*-LAMP assay was confirmed by setting the different reaction temperatures (61–68°C, with 1°C intervals) and using *C. albicans* reference strain (ATCC10231) template DNA (10 pg/μl). Amplification mixtures containing 1 μl of *C. tropicalis* and *K. pneumoniae* template were used as NC, and 1 μl of DW was used as a blank control. The assay was performed according to the standard LAMP assay and was monitored using the Loopamp Realtime Turbidimeter LA-500 (Eiken Chemical Co., Ltd., Japan). Moreover, the threshold value (turbidity) was 0.1, and turbidity of >0.1 was considered positive amplification (Li et al., 2019).

### Sensitivity of the *C. albicans*-LAMP-LFB assays

The genomic DNA templates from *C. albicans* (ATCC10231) pure culture were serially diluted 10-fold (10 ng, 10 pg, 1 pg, 100 fg, 10 fg, 1 fg, and 100 ag per microliter) to confirm the limit of detection (LoD) and to test the analytical sensitivity of LAMP-LFB assay. The LAMP-LFB reactions were carried out under the standard conditions described above, and the results were examined using a VDR and LFB. The LoD of the LAMP-LFB assay was verified as the last dilution of each positive test. Each dilution was tested at least three times.

### Reaction time optimization for *C. albicans-*LAMP assay

Four amplification reaction times (30, 40, 50, and 60 min, with 10 min intervals) were evaluated to optimize the amplification reaction time of *C. albicans*-LAMP. The *C. albicans*-LAMP reactions were performed under standard conditions, and the results were reported using LFB. Each amplification time was repeated three times.

### Specificity of the *C. albicans*-LAMP-LFB assays

Genomic DNA (at least 10 ng/μl) was detected from 16 *C. albicans* strains, 27 non-*C. albicans* fungal strains and four bacterial strains according to the optimal amplification temperature and time to evaluate the specificity of *C. albicans*-LAMP-LFB assay (Table 2). All LAMP results were verified using LFB. Each sample was analyzed thrice independently.

### Application of *C. albicans*-lamp-LFB assay in clinical samples

A total of 330 clinical samples (including 30 whole blood, 100 middle segment urine, and 200 sputum samples) were collected from the First People’s Hospital of Guiyang suspected of having Candida infection to assess the applicability of the *C. albicans-*LAMP-LFB assay. All clinical samples were detected for *C. albicans* using traditional clinical cultural-based and *C. albicans*-LAMP-LFB methods. The routine diagnosis of *Candida* species in the laboratory was carried out using diagnostic methods such as CHROMagar *Candida* and biochemical identification. The *C. albicans-*LAMP-LFB detection was performed as described above.

## Results

### Detection and confirmation of *C. albicans-*LAMP products

**FIGURE 2:**
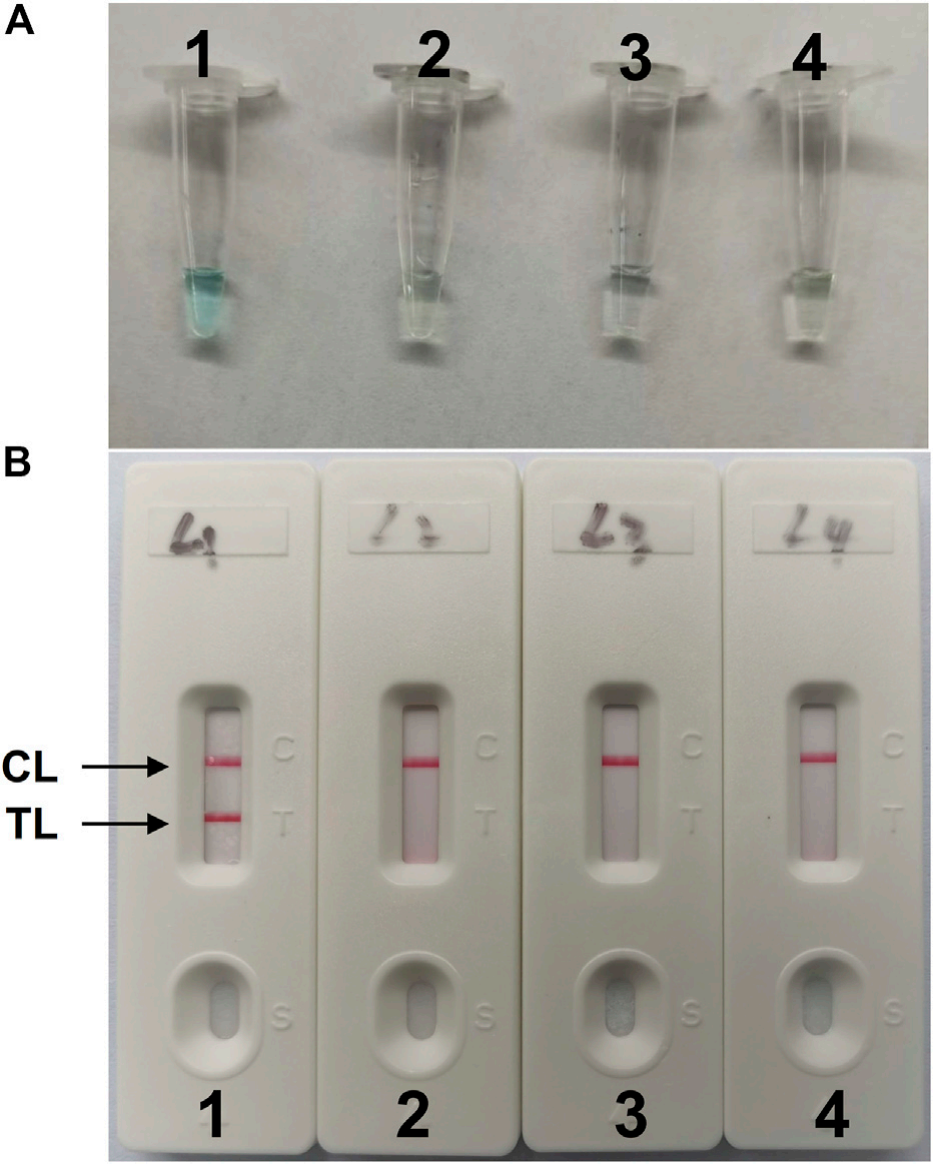
Detection and validation of (C) *albicans*-LAMP products **(A)**, Color change of *C. albicans*-LAMP tubes; **(B)**, biosensor applied for visual detection of *C. albicans*-LAMP products. Tube A1 (biosensor B1), positive amplification; tube A2 (biosensor B2), negative amplification (*Candida tropicalis*), tube A3 (biosensor B3), negative amplification (*Klebsiella pneumoniae*), tube A4 (biosensor B4), negative control (DW); TL, test line; CL, control line.

The LAMP amplification reaction was incubated for 40 min at a constant temperature of 64°C to confirm the availability of *C. albicans*-LAMP primers. Then, the *C. albicans*-LAMP products were examined using the VDR and LFB methods. The *C. albicans*-LAMP amplified products appeared bright green under visible light, while the negative samples and blank control reactions remained colorless (Figure 2A). *C. albicans*-LAMP amplification was further confirmed by the appearance of two crimson red bands (CL and TL) in LFB, while the negatives samples and blank control only appeared as a crimson red line (CL) in LFB (Figure 2B). Therefore, the results suggested that the LAMP primer set targeting the ITS2 gene was valid for establishing the LAMP-LFB assay for *C. albicans* detection.

### Optimal temperature of *C. albicans-*LAMP assay

**FIGURE 3:**
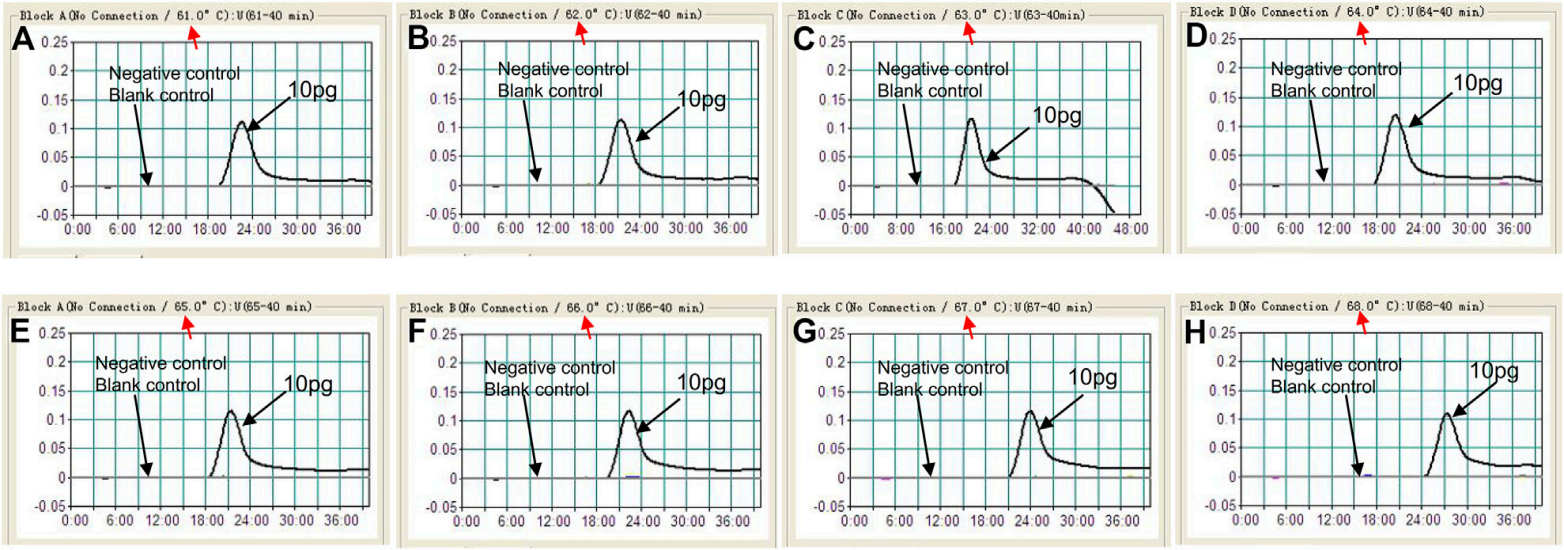
Optimization of amplification temperature for the *C. albicans*-LAMP primer set. The LAMP reactions for detection of *C. albicans* were monitored through realtime turbidity (LA-500), and the corresponding curves of concentrations of templates were marked in the figures. Eight kinetic graphs **(A–H)** were generated at various temperatures (61–68°C, 1°C intervals) with target pathogens DNA at the level of 10 pg per tube (The threshold value was 0.1 and the turbidity of >0.1 was considered to be positive). The graphs from B (62°C) to E (65°C) showed robust reaction. The optimal amplification temperature was 64°C.

The optimization test was performed to obtain the optimal reaction temperature for the *C. albicans*-LAMP experiment by setting a series of amplification temperatures (ranging from 61 to 68°C, with 1°C interval). The results were monitored using real-time turbidity measurements. The kinetics graphs were produced from the eight temperatures, with the faster reactions obtained for *C. albicans*-LAMP reactions at approach temperature of 62–65°C (Figure 3). Hence, we selected 64°C as the optimal amplification temperature for the *C. albicans-*LAMP-LFB assay.

### Sensitivity of *C. albicans-*lamp-LFB detection

**FIGURE 4:**
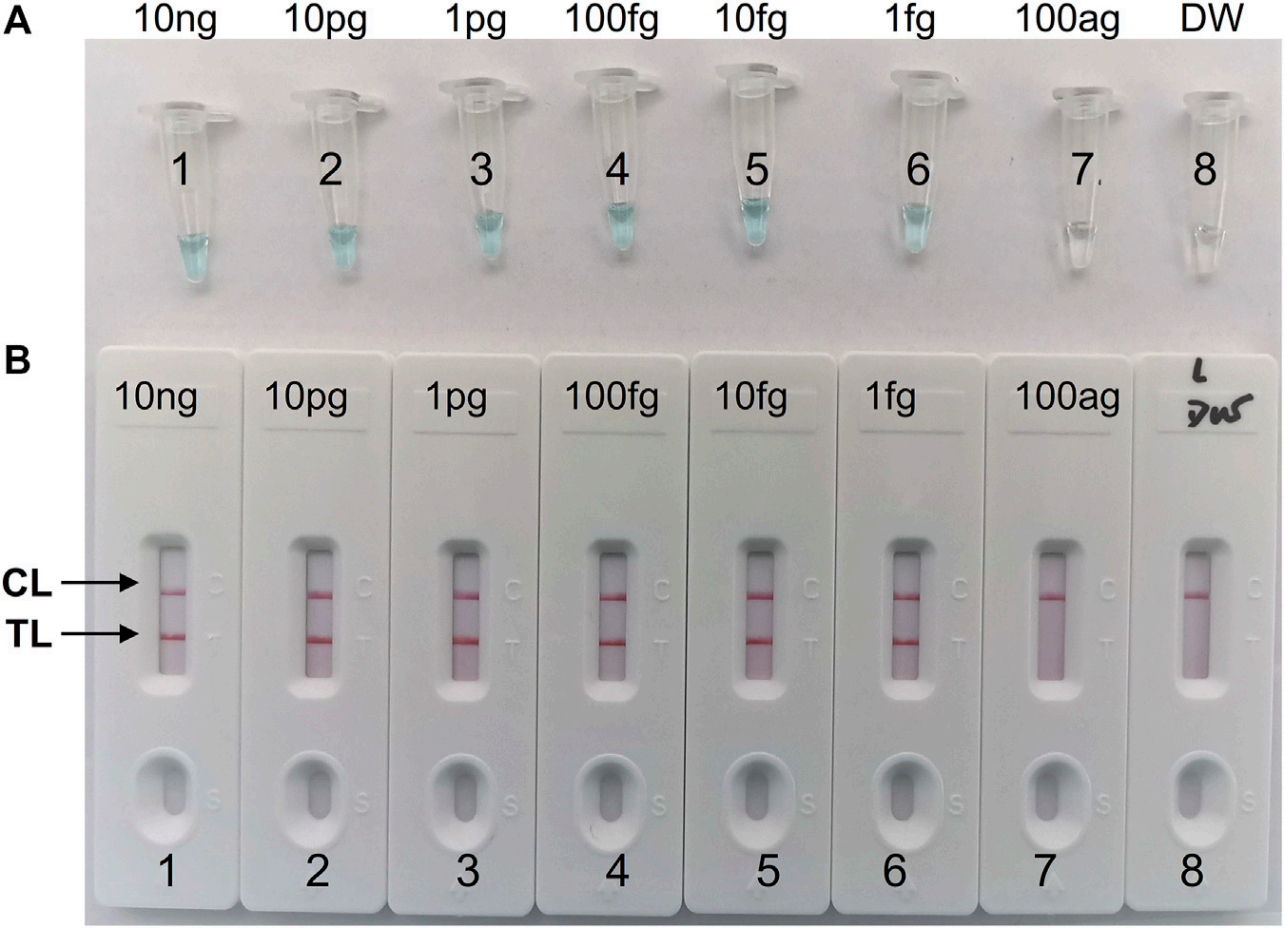
Sensitivity analysis of the *C*. *albicans*-LAMP-LFB assay with serial dilutions of *C*. *albicans* genomic DNA concentrations. Two detection methods involving a colorimetric indictor (VDR) **(A)** and LFB **(B)** were used to analyze the LAMP products. The genomic DNA was serially diluted (10 ng, 10 pg, 1 pg, 100 fg, 10 fg, 1 fg, and 100 ag per microliter) and subjected to standard LAMP. Tubes A1-7 (Biosensors B1-7), *C*. *albicans* strain (ATCC 10231) genomic templates (10 ng-100 ag); Tube A8 (Biosensor B8), blank control (DW), respectively. The LoD of *C. albicans*-LAMP was one fg per reaction.

In our study, repeated detection of serial dilutions of *C. albicans* genomic DNA confirmed the sensitivity of the *C. albicans*-LAMP-LFB method. The *C. albicans*-LAMP-LFB assay LoD of template DNA was 1 fg/μl. Visual inspection of LAMP products with VDR reagents (Figure 4A) revealed CL and TL lines (red) on LFB, indicating positive amplification of the ITS2 gene (Figure 4B). The biosensors of the blank controls displayed only the CL line.

### Optimal time of *C. albicans -*lamp-LFB assay

**FIGURE 5:**
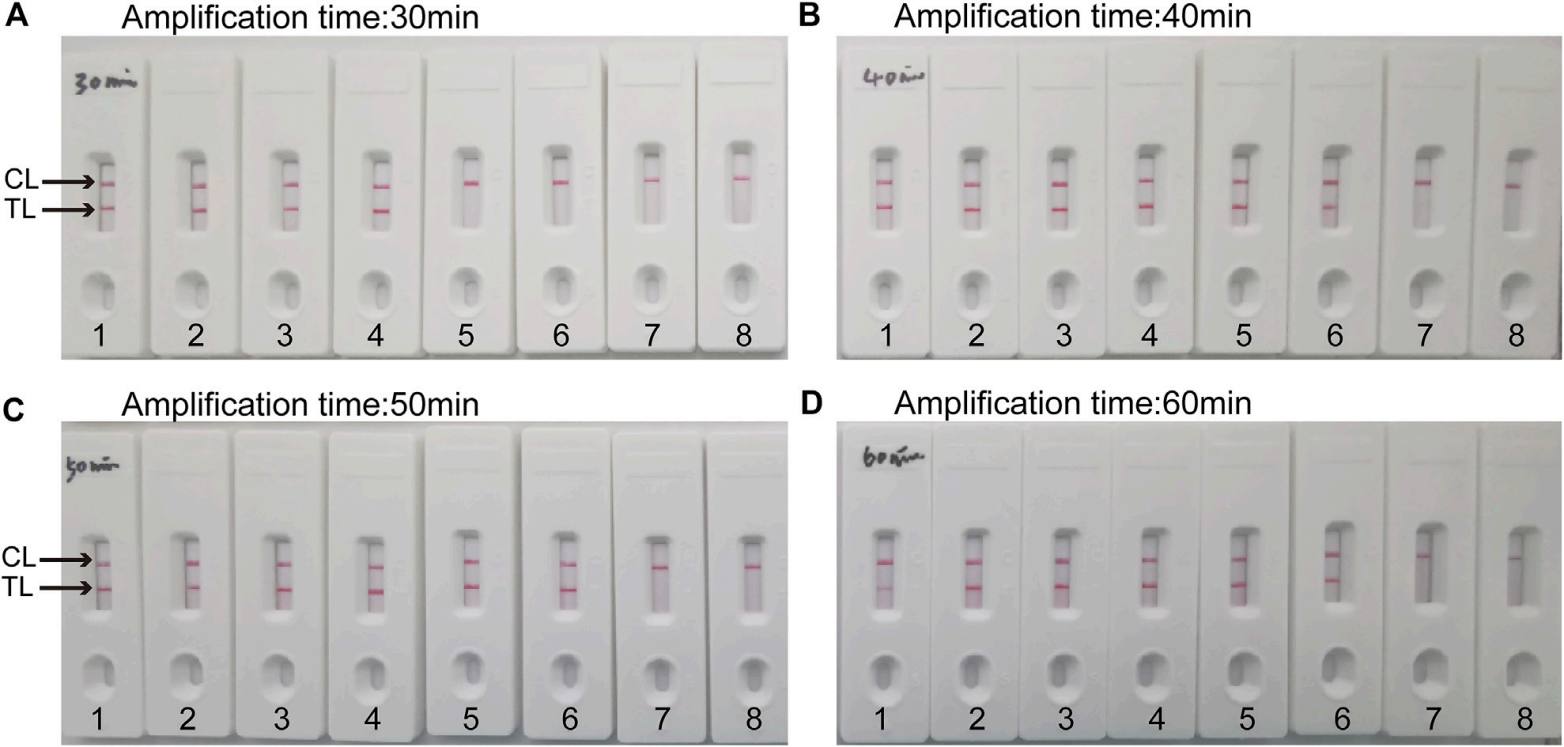
Optimal duration of time required for *C*. *albicans*-LAMP-LFB assay Four amplification times **(A)**, 30 min; **(B)**, 40 min; **(C)**, 50 min; **(D)**, 60 min) were evaluated at 64 °C. Biosensors 1, 2, 3, 4, 5, 6, 7, and eight represent genomic DNA (*C.albicans* (ATCC 10231)) levels of 10 ng, 10 pg, 1 pg, 100 fg, 10 fg, one fg and 100 ag target template per reaction and blank control (DW), respectively. The optimal time was showed when the amplification lasted for 40 min **(B)**.

**FIGURE 6:**
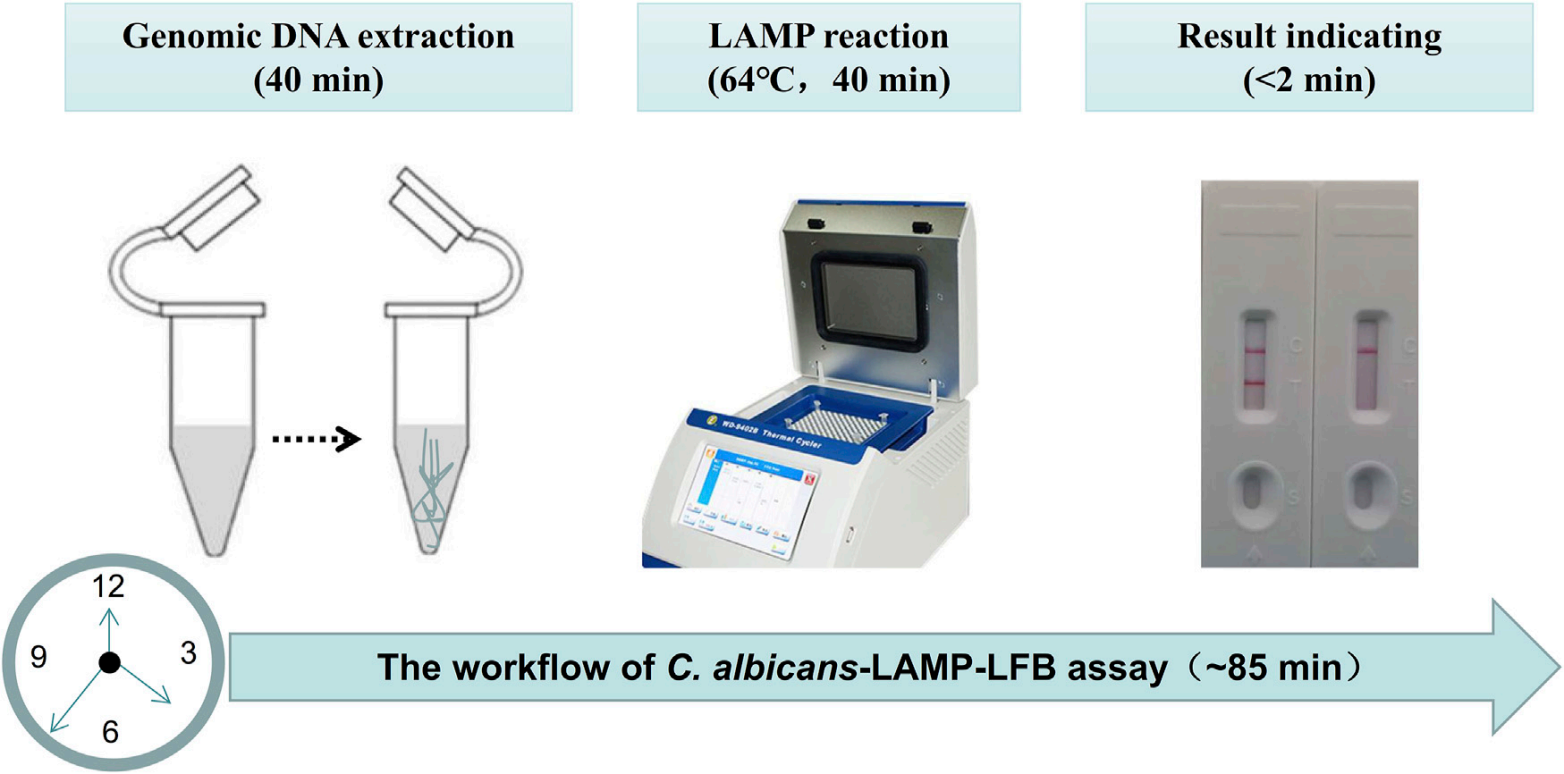
The workflow of *C. albicans*-LAMP-LFB assay. Three steps, including Genomic DNA extraction (40 min), LAMP reaction (40 min) and result reporting (<2 min), are required for the *C. albicans*-LAMP-LFB assay, and the total procedure can be completed within 85 min.

Four reaction time intervals (30, 40, 50, and 60 min) were tested at 64°C to obtain an optimal reaction time for *C. albicans*-LAMP-LFB. The *C. albicans*-LAMP products were identified through VDR and LFB methods. The results confirmed that the *C. albicans* (ATCC 10231) genomic DNA template was detected at the LoD level (1 fg) when the *C. albicans*-LAMP reaction lasted for 40 and 50 min at 64°C (Figure 5). There is no significant difference between the 40 min and 50 min lines. Therefore, a response time of 40 min was chosen for the LAMP-LFB test of *C. albicans*. Incubating *C. albicans-*LAMP-LFB at 64°C for 40 min was the optimal time. Thus, the whole diagnostic procedure of the *C. albicans*-LAMP-LFB assay, including Genomic DNA extraction (40 min), LAMP reaction (40 min) and result indicating (<2 min), can be completed within 85 min (Figure 6).

### Specificity of *C. albicans-*lamp-LFB detection

**FIGURE 7:**
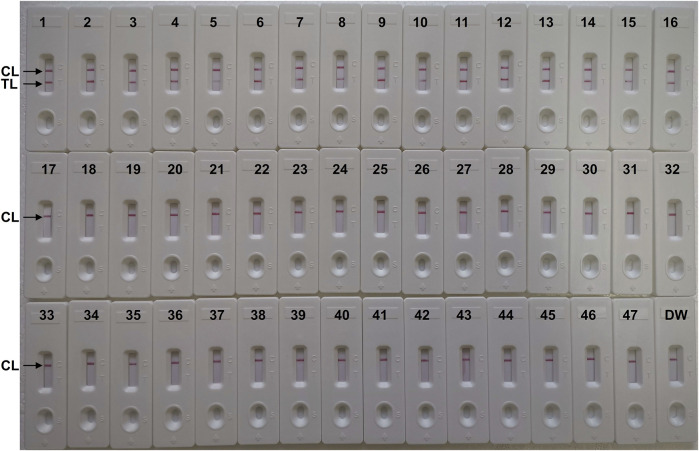
The specificity of *C. albicans*-LAMP-LFB assay for different strains. The *C. albicans*-LAMP-LFB assay was evaluated with different strains genomic DNA as templates. Two crimson lines (TL and CL) were appeared on the LFB, indicating positive results for the *C. albicans* isolates. Only a visible red line (CL) at the detection zone of LFB, reporting the negative results for non-*C. albicans* strains and blank control (DW). Biosensor1, Positive control (*C. albicans* ATCC 10231), Biosensors 2-16, isolated *C. albicans* strains; Biosensor 17, *Candida dubliniensis*; Biosensor18, *Candida tropicalis* (ATCC 13803); Biosensors19-23, isolated *Candida tropicalis* strains; Biosensors 24-28, isolated *Candida krusei* strains; Biosensors 29-33, isolated *Candida glabrata* strains; Biosensors 34-38, isolated *Candida parapsilosis* strains; Biosensor 39, *Cryptococcus neoformans*; Biosensor 40, *Penicillium mameffei*; Biosensor 41, *Candida stellatoidea*; Biosensor 42, *Candida guilliermondi*; Biosensor 43, *Aspergillus flavus*; Biosensor 44, *Staphylococcus aureus*; Biosensor 45, *Klebsiella pneumoniae*; Biosensor 46, *Pseudomonas aeruginosa*; Biosensor 47, *Acinetobacter baumannii*; Biosensor 48, blank control (DW).

The specificity of the *C. albicans*-LAMP-LFB assay was evaluated using DNA from 47 strains, including 1 *C. albicans* reference strain (ATCC10231), 15 *C. albicans* isolated strains, 27 non-*C. albicans* fungal strains and four bacterial strains (Table 2). Figure 7 illustrates that the *C. albicans*-LAMP-LFB assay detected all *C. albicans* strains while not detecting non-*C. albicans* fungal or bacterial strains. Hence, the *C. albicans*-LAMP-LFB assay can accurately differentiate *C. albicans* from other microbes.

### Application of the LAMP-LFB assay in clinical samples for *C. albicans* detection

To verify the practical application of *C. albicans*-LAMP-LFB as a valuable tool for target pathogen detection. Traditional culture method identified *C. albicans* in 62 (18.8%) of the 330 samples (including two of the 30 whole blood, 18 of the 100 middle segment urine, and 42 of the 200 sputum samples), while the remaining 268 samples contained other bacteria or were microbe-free. The *C. albicans*-LAMP-LFB assay results were consistent with the traditional cultivation detection results. Table 3 suggests that the *C. albicans*-LAMP-LFB assay established in the current study could be used as an advanced tool to detect *C. albicans* in clinical samples.

**TABLE 3:** Comparison of conventional culture and *C. albicans*-LAMP-LFB methods to identify *C. albicans* in clinical samples.

Detection methods^a^	Blood samples (*n* = 30)		Middle segment urine (*n* = 100)		Sputum samples (*n* = 200)		Time consumption	Sensitivity (%)	Specificity (%)
	Positive	Negative	Positive	Negative	Positive	Negative			
Culture-based assay	2	28	18	82	42	158	At least 72 h	100	100
LAMP-LFB assay	2	28	18	82	42	158	Within 85 min	100	100

^a^ LAMP, loop-mediated isothermal amplification; LFB, lateral flow biosensor.

## Discussion

*C. albicans* is a common but important opportunistic pathogen that frequently causes superficial and deep fungal infections and is primarily responsible for invasive candidiasis such as pyelonephritis, endocarditis, and candidemia (Gow and Yadav, 2017). It is also considered the fourth leading cause of hospital bloodstream infection, with unacceptably high mortality rates (Dadar et al., 2018). Currently, invasive candidiasis is difficult to diagnose and has a poor prognosis, posing a huge burden on public health worldwide (Casadevall, 2019). However, antifungal drugs can be employed to treat fungal infections; early and accurate diagnosis results in effective treatment outcomes. Moreover, *C. albicans* has increased drug resistance by forming biofilms, making antifungal treatments more intractable (Ben-Ami, 2018). Therefore, developing a rapid and accurate diagnostic method for *C. albicans* is a high priority to improve patient quality of life, reduce patient mortality and drug resistance. Rapid pathogen identification is important in controlling and managing invasive candidiasis infection (McCarty and Pappas, 2015). However, current methods for diagnosing *C. albicans* infection are time-consuming, cumbersome, and uneconomical. Hence, an accurate, rapid, simple, and economical method for detecting *C. albicans* is urgently needed. This report combined classic LAMP with LFB to detect *C. albicans.* LAMP primer sets, including two inner primers (FIP and BIP), two outer primers (F3 and B3), and two loop primers (LF and LB), were designed per LAMP rules to recognize the sequence of the ITS2 gene of *C. albicans* with a high degree of specificity (Notomi et al., 2000). The entire procedure could be completed within 85 min, including specimen processing (40 min), isothermal reaction (40 min), and result reporting (within 2 min). Importantly, we could visually evaluate the *C. albicans*-LAMP results based on the bands on the LFB within 2 min; even non-experts can correctly interpret the results.

LAMP is more convenient and cost-effective than traditional methods such as direct microscopy, but it is not sensitive enough to distinguish all *Candida* species. Thus, the diagnosis can be ambiguous, delaying precise treatment. Although the CHROMagar *Candida* method is simple, it is time-consuming (maybe spend 3 days or more). Moreover, it is impossible to distinguish all *Candida* species, especially due to the color similarity of the colonies, such as *Candida dubliniensis* (Ells et al., 2011)*.* Furthermore, compared to other molecular diagnostic methods that are more accurate and time efficient than traditional culture methods, real-time PCR, for example, requires professionals to operate special instruments to change the temperature to complete the experiment, making it cumbersome and uneconomical, limiting its application, especially in areas where resources are scarce (Asadzadeh et al., 2018). In this reaction system, the optimal LAMP conditions were 64°C for 40 min; a very simple and inexpensive device, such as a water bath or a heater, is sufficient to keep the reaction temperature at 64°C for 40 min. It reveals that LAMP is time-saving, economical, and easy to operate. We also compared LAMP to other isothermal amplification assays, for example, Multiple cross displacement amplification (MCDA), for *C. albicans* detection (Zhao et al., 2019). In Zhao’s report, the LoD to detect *C. albicans* was 200 fg, while in our study, the sensitivity was one fg of genomic DNA template from *C. albicans* pure cultures. The results demonstrated that LAMP was more sensitive than MCDA in our study. In addition, the LoD of the *C. albicans*-LAMP-LFB method developed in this report was 1 fg/μl of *C. albicans* genomic DNA, which was 10 times more sensitive that of the conventional LAMP added SYBR Green I amplification indicator to the reaction mixture in the experiment (Fallahi et al., 2020). Moreover, we demonstrated the assay specificity of the *C. albicans*-LAMP method using DNA from 47 strains, including one *C. albicans* reference strain (ATCC10231), 15 *C. albicans* isolated strains, 27 non-*C. albicans* fungal strains and four bacterial strains. No cross-reactions to non-*albicans* strains were obtained, and the analytical specificity of the LAMP-LFB assay is 100%; therefore, the LAMP-LFB assay could be employed to detect *C. albican* with high specificity.

The LAMP assay has been used to detect various pathogens, including *C. albicans* (Wang et al., 2017; Yamamoto et al., 2018; Fallahi et al., 2020; Zhu et al., 2020). Unfortunately, traditional LAMP assay results were tedious to interpret. It requires special materials and instruments, such as agarose gel electrophoresis, real-time turbidity equipment, and an SYBR green I color indicator (Hill et al., 2018; Zhao et al., 2020). Worse, it requires a professional to operate. Hence, the factors mentioned above limited their point-of-care testing. This study used VDR and LFB methods to detect LAMP products, avoiding the shortcomings of traditional LAMP assays. The color of the amplified products effectively changed from colorless to light green in the VDR assay, while the negative and blank controls remained colorless. Moreover, CL and TL appeared on the LFB, revealing positive LAMP results for the ITS2 gene. However, only the CL line was observed on the biosensor for the blank controls. These two methods are visual and very simple; non-specialists can interpret the results accurately. Although the VDR assay can detect LAMP products, the color is fuzzy when the product concentration is low, which may lead to interpretation errors (Chen et al., 2021). So, the biosensor LFB is considered a suitable method for monitoring *C. albicans-*LAMP products due to its high sensitivity, simplicity of operation, and cost savings. The total cost of one test, including fungi genomic DNA extraction (approximately $1 USD), *C. albicans*-LAMP reaction (approximately $3.5 USD) and LFB reporting (approximately $2 USD), is estimated to be $6.5 USD, which is cheaper than conventional PCR-based methods. Combined with the elimination of labor costs because of the requirements for trained personnel in a certified laboratory, the *C. albicans*-LAMP-LFB assay becomes more cost-effective.

We evaluated the clinical application of *C. albicans*-LAMP-LFB using 330 clinical samples (including 30 whole blood, 100 middle segment urine, and 200 sputum samples); the LAMP-LFB assay identified all *C. albicans-*positive (62/330) samples. The diagnostic accuracy was 100% compared to the traditional clinical cultural-based methods. The results suggested that this assay can be used as a diagnostic tool for the rapid, accurate, sensitive, and specific detection of *C. albicans* strains. It is especially useful in implementing point-of-care testing, which is more conducive to developing resource-poor areas. Our study has some limitations LAMP-LFB assay is a qualitative test without quantitative detection. Hence, it is difficult to determine whether it is a pathogen or a colonizing bacterium. Therefore, in the future research, this method should focus on the detection of blood, middle segment urine, bronchoalveolar lavage fluid and aseptic body fluid, and try to avoid the detection of sputum samples, which is conducive to the correct use of antifungal drugs. In addition, LAMP is an isothermal amplification technique that requires multiple primer pairs; contamination can easily occur, leading to incorrect results. Therefore, we will address the above deficiencies in subsequent studies.

## Conclusion

In conclusion, a visual, rapid, simple, sensitive, and cost-saving *C. albicans*-LAMP-LFB assay based on the ITS2 gene was successfully devised for identifying *C. albicans* agents in the current study. The *C. albicans*-LAMP-LFB assay showed high sensitivity and high specificity, which could successfully detect *C. albicans* isolates, and had the LoD of 1 fg genomic DNA template per tube. Besides, the clinical effectiveness of this assay was successfully upheld by clinical samples, including whole blood, middle segment urine, and sputum samples. Hence, our results indicate that *C. albicans*-LAMP-LFB strategy is an effective tool for *C. albicans* rapid detection in clinical samples, especially in resource-limited settings.

## Data Availability

The original contributions presented in the study are included in the article/Supplementary Material, further inquiries can be directed to the corresponding authors.
